# Assessment of fermented vegetable wastes in Nile tilapia diets: impacts on fish performance, amino acids profile, health, and intestine histomorphology

**DOI:** 10.1038/s41598-026-56610-6

**Published:** 2026-06-16

**Authors:** Norhan E. Saleh, Ahmed E. Sallam, Fady R. Michael, Heba H. Abdel-Mohsen, Hebatollah M. Almisherfi, Elham A. Wassef

**Affiliations:** National Institute of Oceanography and Fisheries, Cairo, Egypt

**Keywords:** Aquafeed ingredient, Anti-nutritional factors, Solid state fermentation, Fish health, Sustainable aquaculture, Biochemistry, Microbiology, Physiology, Zoology

## Abstract

This study assessed the effects of incorporating fermented vegetable wastes (FVW), with *Bacillus subtilis* and *Saccharomyces cerevisiae*, in Nile tilapia (*Oreochromis niloticus*) diets at 0, 10, 20, and 30% inclusion levels (FVW0, FVW1, FVW2, and FVW3, respectively) on fish growth, feed utility, amino acids profile, health, nutrient utilization and intestine histomorphology. The results indicated 100% survival rates for all experimental groups. Furthermore, based on WG and SGR recorded values, fish growth was insignificantly changed among all fish groups (*P* > 0.05). Feed intake was significantly higher in FVW3 and FVW2 compared to other groups, however, feed utilization efficiency was not varied among all experimental groups depending on FCR and PER values. Carcass biochemical analysis showed that only ash content was significantly different among dietary groups as it was higher in FVW1 and FVW2 compared with the other two groups. The results illustrated an increase in most of the essential amino acids (EAAs) percent in the experimental groups relative to the control. Complete blood count revealed that red blood cells count remained insignificantly different among all groups, however, FVW1 showed a significantly lower hemoglobin content compared to the control and other groups. White blood cells count was significantly the highest in FVW2. Aspartate aminotransferase (AST) concentration was significantly elevated in both FVW2 and FVW3 groups compared to control, however, alkaline phosphatase (ALP) concentration was significantly decreased in these two experimental groups. The highest alanine aminotransferase (ALT) concentration was recorded in FVW3. Total protein and globulin values were significantly increased in all tested groups relative to the control. Proteases activity was propositionally increased with increasing FVW inclusion level, however, both lipase and amylase activities were significantly decreased in the treated groups relative to the control. Histomorphological inspection of fish distal intestine revealed normal intestinal architecture with enhanced villi development and goblet cell density in FVW-fed fish, particularly at 20% inclusion. The present results indicate that FVW can be safely included in Nile tilapia diets up to 30%.

## Introduction

Aquaculture plays a fundamental role in global food security, however, the sector faces increasing pressure to find affordable and environmentally sustainable protein sources^1^. Conventional feed ingredients, such as fishmeal and soybean meal, are becoming economically and ecologically unsustainable due to rising costs and intensive requirements^2^. Consequently, there is an urgent need to explore alternative feedstuff that aligns with circular economy principle. Vegetable peels, often discarded as agricultural waste, represent a promising underutilized resource. These by-products are valuable sources of nutrients and functional components^3^. They are rich in vitamins, minerals, dietary fiber, and phenolic compounds, offering a double benefit; reducing both organic wastes accumulation and greenhouse gas emissions from landfills while lowering dependency on traditional feed ingredients^4^.

Vegetable by-products meal is not a well-researched ingredient in freshwater fish nutrition although it has beneficial bioactive compounds, including antioxidants and anti-inflammatory agents^5^. Studies have demonstrated that pea meal may be used as a total or partial replacement for fishmeal in the diets of farmed fish, without compromising the ability of the fish to thrive including Asian sea bass (*Lates calcarifer*), Atlantic salmon (*Salmo salar*), and Rainbow trout (*Oncorhynchus mykiss*)^6–8^; respectively. According to Serra et al.^9^, pea pods meal is a valuable alternative to fishmeal, with high potential as it is able to decrease production costs and environmentally friendly. According to Tewari et al.^10^, adding 20% pea peel powder to the diet of common carp (*Cyprinus carpio*) resulted in increased weight gain, specific growth rate, and a lower feed conversion ratio, indicating the effectiveness of this by-product in improving growth performance. Similar growth and survival rates were shown by Nile tilapia fed diets that included pea meal as a partial replacement for fishmeal^11–13^. However, according to Magbanua and Ragaza^14^, the optimal inclusion level for pea peels meal has been reported differently by various researchers, adding to the data gap.

Despite these positive features, the direct incorporation of vegetable by-products into aquafeed is restricted by the existence of anti-nutritional factors (ANFs) which are indigestible components that reduced nutrient bioavailability. Accordingly, using these wastes in aquafeed needs effective processing methods to improve their nutritional value, get rid of the anti-nutritional substances, optimize resource utilization, and ensure their safety^3^. Mostly, the existence of ANFs such as; phytate, trypsin inhibitors, and lectins, usually affects palatability and restricts digestion, dietary efficient utilization, thus inducing variation in growth performance, immunity, and lead to tissue inflammation^15^ (Dossou et al., 2018). Demirci^16^ mentioned that a higher pea protein supplementation, in rainbow trout diets, caused severe histopathological changes in the liver.

To overcome these mentioned constraints, solid-state fermentation (SSF) has developed as a strategic bioprocessing technique. By utilizing microorganisms to break down complex polysaccharides and proteins, SSF enhances the nutrient digestibility, palatability, and mineral bioavailability while mitigating ANFs^17–19^. According to Oluremi and Okhonlaye^20^, green peas contain ANFs like enzymes inhibitors, phytates, oxalates, saponins and polyphenolic compounds, all of them limit peas dietary utilization. They evaluated the effect of fermentation on the antinutrients content of green pea. They mentioned that fermentation reduced the ANFs of the fermented sample from 32.18 mg/g, 4.14 mg/g, 1.62 mg/g, 51.08 mg/g and 36.37 mg/g in the raw sample to 26.27 mg/g, 0.48 mg/g, 0.27 mg/g, 7.82 mg/g and 24.07 mg/g, respectively. Furthermore, Boroojeni et al.^21^ measured the anti-nutritional factors in pea by-products before and after solid-state fermentation, concluding that fermentation enhanced their nutritional quality by reducing these ANFs by 1.9–77.5%, with the extent of reduction varying according to the specific compound. Moreover, these results are aligned with Liu et al.^22^ who mentioned that, fermentation of pea peels increases their nutritional content. Siddik et al.^23^ suggest that fermented plant-based ingredients can significantly improve fish growth performance and health status. Despite these advantages, optimal inclusion levels and the specific effects of fermented vegetable wastes on Nile tilapia (*Oreochromis niloticus*) physiology and health remain insufficiently characterized. Therefore, this study aims to evaluate the efficacy of incorporating fermented vegetable wastes as a sustainable ingredient in Nile tilapia diets. The current study investigates the effects of fermented vegetable by-products on fish growth performance, feed utilization, amino acid composition, health, and intestine histomorphology. This research provides a viable strategy for enhancing economic efficiency and environmental stewardship in sustainable aquaculture.

## Methodology

This proposal was reviewed and approved by the Institutional Animal Care and Use Committee of National Institute of Oceanography and Fisheries (NIOF-IACUC) under the approval code: NIOF-AQ4-F-25-R-039. Authors confirm that the experiment was performed in accordance with relevant guidelines and regulations and they complied with the RRIVE guidelines (https://arriveguidelines.org).

### Fermented vegetable by-products meal (FVW)

Vegetable wastes (VW), mainly pea peels with minor proportions of potato, carrot, beetroot, green okra, and zucchini by-products, were obtained from a local market (Alexandria, Egypt). The discards were chopped into ~ 5 × 5 cm pieces, dried at 50 °C for 17 h in an oven, and ground into a fine powder.

For solid-state fermentation (SSF), 200 g of substrate was placed in 1000 mL conical flasks, and moisture content was adjusted to 35% (w/w) using sterile distilled water. Flasks were autoclaved at 121 °C for 15 min and cooled to room temperature. The cooled substrate was inoculated with 1% (w/w) microbial inoculum containing *Saccharomyces cerevisiae* (3.7 × 10⁹ CFU/g) and *Bacillus subtilis* (7.7 × 10⁸ CFU/g). Fermentation was carried out at 37 °C for 5 days under static conditions.

The fermented product (FVW) was dried at 55 °C for 48 h and stored at 4 °C until use. *S. cerevisiae* was selected for its capacity to secrete cellulase and amylase for agricultural waste conversion^24^, while *B. subtilis* was chosen for its production of extracellular enzymes including proteases, β-glucanases, α-amylase, and lipolytic enzymes^25^.

### Experimental diets

The biochemical composition of all dietary ingredients before and after fermentation is illustrated in Table 1. Four experimental diets (~ 31% CP) were formulated to include 0, 10, 20, and 30% FVW and assigned as FVW0 (control), FVW1, FVW2, and FVW3, respectively (Table 2). All the ingredients were finely ground, thoroughly mixed, pelleted at 3 mm using a laboratory pellet mill, dried at 50 °C, and then kept at − 20 °C until further use.

**Table 1:** Biochemical composition of the vegetable wastes (VW), fermented vegetable wastes (FVW), and other ingredients used in the experimental diets.

Biochemical composition (%)	Moisture	Crude protein	Ether extract	Ash	Fiber
Vegetable waste (VW)	89.96	18.13	6.98	15.71	11.50
Fermented vegetable waste (FVW)	91.59	26.21	5.07	13.63	10.56
Soybean meal	8.92	48.40	1.02	6.51	6.02
Wheat middlings	9.15	15.10	3.20	2.40	2.60
Corn meal	7.90	8.80	3.81	1.40	2.20

**Table 2:** Experimental Diets formulation and their biochemical composition (% on dry weight).

Diet formulation (%)	Diets (% dry weight)			
	FVW0	FVW1	FVW2	FVW3
Fish meal	15.0	15.0	15.0	15.0
Soybean meal	32.0	29.3	26.6	23.9
Wheat middling	22.9	20.9	18.9	16.9
Corn meal	11.2	9.3	7.3	5.3
Rice bran	12.6	12.6	12.6	12.6
FVW meal^1^	0.0	6.6	13.3	20.0
Fish oil	2.0	2.0	2.0	2.0
Vegetable oil	1.0	1.0	1.0	1.0
Soy lecithin	1.0	1.0	1.0	1.0
Mono-calcium phosphate	1.2	1.2	1.2	1.2
Vitamins & minerals p remix^2^	0.8	0.8	0.8	0.8
Vitamin C	0.2	0.2	0.2	0.2
Betaine	0.1	0.1	0.1	0.1
Proximate composition (% dry basis)				
Dry matter	91.22	91.00	90.58	91.64
Crude protein	31.49	31.33	31.13	31.28
Crude fiber	4.37	4.81	5.26	5.71
Crude fat	7.71	7.69	7.61	7.55
Ash	7.67	7.80	9.01	9.63
NFE^3^	48.76	48.37	46.99	45.83

All ingredients were purchased from Al-Nahda Feed Company (Kafr Elsheikh, Egypt).

^1^Fermented vegetable wastes (Lab. Made)

^2^Premix (g/kg): MgSO_4_·7H_2_O, 80.0; NaH_2_PO_4_·2H_2_O, 370.0; KCl, 130.0; ferric citrate, 40.0; ZnSO_4_·7H_2_O, 20.0; Ca-lactate, 356.5; CuCl, 0.2; AlCl_3_·6H2O, 0.15; KI, 0.15; Na_2_Se_2_O_3_, 0.01; MnSO_4_·H_2_O, 2.0; CoCl_2_·6H_2_O, 1.0. L-ascorbic acid, 121.2; DL-α-tocopheryl acetate, 18.8; thiamin hydrochloride, 2.7; riboflavin, 9.1; pyridoxine hydrochloride, 1.8; niacin, 36.4; Ca-D-pantothenate, 12.7; myo-inositol, 181.8; D-biotin, 0.27; folic acid, 0.68; p-aminobenzoic acid, 18.2; menadione, 1.8; retinyl acetate, 0.73; cholecalciferol, 0.003; cyanocobalamin, 0.003.

^3^Nitrogen-free extract (NFE) = 100 − (crude protein + crude lipid + ash + crude fiber).

The proximate analysis of the feed ingredients and experimental diets was completed in triplicate following AOAC^26^ procedures. Protein content was determined using the Kjeldahl method, lipid content by the Soxhlet apparatus, moisture content by heating the feed ingredients and the formulated diets at 60 °C in an oven until a constant weight was attained, and furthermore, the ash content was determined by burning the samples at 550 °C for 6 h in the muffle furnace.

### Fish rearing

A total of 500 Nile tilapia (*Oreochromis niloticus*) fingerlings (3.50 ± 0.08 g) were bought from a private fish farm, Kafr El-Sheikh. The fingerlings were acclimatized in five tanks (0.5 m^3^) for 14 days and fed on a commercial diet twice daily (30% CP and 8% CL). At the beginning of the experiment, 360 healthy and uniform fish were randomly chosen and distributed into 12 tanks, 100 L each (30 fish/tank). The fish were feed to visual satiation three times daily (9.00 am, 1.00 pm and 4.00 pm) on the designed experimental diets for 60 days. Water quality was controlled using multiparameter probe. Temperature was 26–27 °C, DO range was 6.9–7.2 mg/L, pH was 7.2–7.3, ammonia was 0.10–0.12 mg/L, and nitrite concentration 0.06–0.10 mg/L.

At the terminal of the experiment, ten fish from each tank were killed for the proximal analysis and amino acids composition. Euthanasia was carried out using an overdose of clove oil at a concentration of 250 mg/L in water^27^. Fish were immersed in the solution until cessation of opercular movement for a minimum of 10 min, confirming death. The proximate analysis of the experimental fish was completed on triplicate basis following AOAC^26^ procedures.

### Fish growth and feed utility

The fish were counted and weighed per tank, at the terminal of the experiment after 24 h. of the last meal. The growth and feed utilization efficacy were calculated as following: Weight gain (WG, g) = Wf – Wi, where Wf is the final fish weight (g) and Wi is the initial fish weight (g); feed conversion ratio (FCR) = feed consumption (g)/WG ; specific growth rate (SGR; %/day) = 100 [(Ln Wf) – (Ln Wi)]/60; Protein efficiency ratio (PER) = WG (g)/protein consumed (g); Fish survival (SR, %) = 100 (final fish count/initial fish count). Condition factor (K) = 100 (TW/L^3^) where TW= total fish weight (g), L= total fish length (cm). Hepatosomatic index (HSI) = 100 [liver weight (g)/TW]; Viscerosomatic index (VSI) = 100 [viscera weight (g)/TW].

### The hematological measurements

At the end of the experiment, 5 fish / tank (15 fish/treatment) were anaesthetized with clove oil (20 mg/L) for 5 min^28^ until they loss of equilibrium and opercular movement slowed. Approximately 3.0 mL of blood was drawn from the caudal vein using a sterile heparinized syringe. All procedures were performed under strict aseptic conditions to minimize stress.

The complete blood count (CBC) of each blood sample was determined using a Fully Automatic Blood Cell Counter. Assessment of the red blood cell count (RBC, 10^6^ /µl), haemoglobin concentration (Hb, g/dl), hematocrit value (Hct, %), mean corpuscular volume (MCV, Fl), mean corpuscular hemoglobin (MCH, pg), mean corpuscular hemoglobin concentration (MCHC, g/dL), white blood cell count (WBC, 10^3^ /µl), and platelets (10^3^ / µl) were carried out. The differentiation of WBCs was also carried and neutrophil, lymphocytes, monocytes, and eosinophil percent were also quantified.

### Serum biochemistry

Further samples of blood were allowed to coagulate at room temperature and then placed in centrifuge tubes for 10 min at 3000 × *g* at the ambient temperature to separate the sera. For later usage, the sera were stored at – 20 °C after being separated using the centrifugation technique. The blood biomarkers assessment was carried out using colorimetric evaluation utilizing certain commercial kits (Sigma-Aldrich, USA). Serum total protein (g/dL) was determined according to Henry^29^ using the Biuret reaction, where peptide bonds react with copper ions under alkaline conditions to form a violet-colored complex measured at 540 nm. Albumin (g/dL) was quantified by the bromocresol green dye-binding method^30^, forming a green-colored complex measured at 628 nm and globulin (g/dL) was calculated by subtracting albumin from total protein.

Alkaline phosphatase (ALP, U/L) activity was measured using the Belfield and Goldberg method^31^, based on hydrolysis of p-nitrophenyl phosphate to p-nitrophenol in an alkaline medium, with absorbance read at 405 nm. Aspartate aminotransferase (AST) and alanine aminotransferase (ALT) activities in serum were determined according to the colorimetric method of Reitman and Frankel^32^. This assay is based on the transamination reaction that produces oxaloacetate (for AST) or pyruvate (for ALT), which subsequently react with 2,4-dinitrophenylhydrazine to form colored hydrazone derivatives. Absorbance was measured at 505 nm, and enzyme activities were expressed as units per liter (U/L) based on standard curves.

### Serum digestive enzymes

Serum was selected for digestive enzymes analysis as it provides a systemic indicator of metabolic adaptation and has been validated in recent Nile tilapia study^33^. All assays were performed using commercial kits (Sigma-Aldrich, USA) strictly according to the manufacturer’s technical protocols, which explicitly validate serum as an acceptable sample type for direct analysis. Protease activity (Cat. No. PC0100) was assessed by casein hydrolysis; trichloroacetic acid (TCA)-soluble peptides generated by protease activity react with Folin-Ciocalteu’s reagent to produce a blue chromophore measured at 660 nm. One unit of protease is defined as the amount of enzyme that produces color equivalent to 1.0 µmol of tyrosine per minute at pH 7.5 and 37 °C^34^. Lipase activity (Cat. No. MAK046) was measured via a coupled enzyme reaction where lipase hydrolyzes triglycerides to glycerol, quantified calorimetrically at 570 nm. One unit of lipase is defined as the amount of enzyme that generates 1.0 µmol of glycerol per minute at 37 °C. Amylase activity (Cat. No. MAK009) was determined using a coupled enzymatic reaction in which amylase cleaves the substrate ethylidene-pNP-G7, releasing p-nitrophenol measured calorimetrically at 405 nm. One unit of amylase is defined as the amount of enzyme that generates 1.0 µmol of p-nitrophenol per minute at 25 °C.

For all assays, serum samples (25–50 µL) were added directly to assay plates as validated by the manufacturer protocols. Activities were calculated from standard curves provided in each kit and expressed as U/L.

### The amino acids profile

Using a high-performance amino acids analyzer (Biochrom 30), the amino acid composition of the experimental diets and fish (Tables 3 and 6) was ascertained following acid hydrolysis (6 N HCl under reflux for 24 h at 110 °C). Data processing and collecting were done using EZChrom software.

**Table 3:** Amino acid profiles (% of the diet) of the experimental diets fed to Nile tilapia (*O. niloticus*) for 60 days.

Essential amino acids	FVW*	Experimental diets				Requirement**
		FVW0	FVW1	FVW2	FVW3	
Arginine	1.02	1.97	1.92	1.87	1.82	1.68
Histidine	0.43	0.75	0.74	0.72	0.71	0.70
Isoleucine	0.78	1.57	1.53	1.49	1.45	1.15
Leucine	1.21	2.24	2.19	2.14	2.09	2.15
Lysine	1.23	1.93	1.92	1.90	1.89	1.98
Methionine	0.36	0.69	0.69	0.68	0.68	0.10
Phenylalanine	0.96	1.49	1.46	1.43	1.41	1.13
Threonine	0.69	1.26	1.24	1.21	1.19	1.11
Valine	0.90	1.60	1.57	1.54	1.51	1.34
Non-essential amio acids						
Alanine	1.55	3.21	3.34	3.38	3.43	–
Aspartic Acid	1.57	2.95	2.87	2.80	2.72	–
Cystine	0.24	0.46	0.45	0.43	0.41	–
Glutamic Acid	3.19	5.16	5.02	4.88	4.74	–
Glycine	1.02	1.60	1.58	1.57	1.55	–
Proline	0.78	3.52	3.48	3.43	3.38	–
Serine	0.74	1.37	1.34	1.31	1.27	–
Tyrosine	0.57	1.21	1.18	1.15	1.11	–

*FVW: fermented vegetable waste.

**Nile tilapia requirements^35^.

The experimental diets were fulfilled the fish essential amino acids (EAAs) requirements according to Ogunji et al.^35^ except for lysine and leucinee (Table 3), however, according to Santiago and Lovell^36^, all EAAs requirements were fulfilled.

### Histological analysis

At the end of the trial, three fish from each tank were dissected and samples (1 cm) from the distal intestine were collected. Tissues were fixed in 10% neutral buffered formalin, dehydrated in graded ethanol, cleared in xylene, and embedded in paraffin wax. Sections of 5 μm thickness were stained with Hematoxylin and Eosin (H&E). Histological examination was conducted using light microscopy (LEICA, Leica Microsystems AG) and digitally photographed at 400× magnification with Leica digital camera model D-LUX, and processed using LAS EZ imaging software.

### Intestinal histomorphology

Histo-morphometric analysis was performed on nine intestinal sections for each treatment (3 fish/tank). Three parameters were measured: Muscular layer thickness (M) was quantified at three points per section and averaged (µm), villus length (VL: the length from the villus bottom to the tip, µm), and villus width (VW: width at the middle part of the villus, µm). Only complete villi were selected and measured under 400× magnification for each section. Goblet cell density was expressed as cell count per 100 μm of villus epithelium.

### Statistical analysis

Means ± standard error formula was used to express the results. Prior to analysis, data were examined for homogeneity of variances (Levene test) and normal distribution (Shapiro-Wilk test). After that, the data were put through a one-way ANOVA and Duncan’s test to determine whether there were any significant group differences at *P* < 0.05. Version 20 of the SPSS software (SPSS, USA) was used for all statistical analyses.

## Results

### Effects of solid-state fermentation

The solid-state fermentation of the vegetable wastes was significantly increased the wastes protein content from 18.30 to 26.21% with a moderate reduction in lipid, ash, and fiber contents (Table 1).

### Fish growth and feed utility

The growth performance, feed utilization efficiency, and biometric indices results are presented in Table 4. The results showed insignificant difference amongst all dietary groups when considering either WG or SGR. FI values showed significant elevation in FVW2 and FVW3 groups (10.95 & 11.15, respectively) relative to FVW0 group (9.22) (*P* < 0.05), however, FCR values were numerically higher (*P* > 0.05) in these two groups (1.46, 1.66 respectively) compared with FVW0 group (1.24). The most favorable FCR and PER values were observed in FVW1 (1.12 and 2.97, respectively) (*P* > 0.05).

**Table 4:** Growth performance, feed utilization, and somatic indices (mean ± SE) of Nile tilapia (*O. niloticus*) fed the experimental diets for 60 days.

Parameter	FVW0	FVW1	FVW2	FVW3	*P* value
IW	8.71 ± 0.01	8.74 ± 0.03	8.76 ± 0.04	8.73 ± 0.06	0.814
FW	16.14 ± 0.33	15.57 ± 0.63	16.28 ± 0.21	15.76 ± 0.86	0.797
WG	7.41 ± 0.43	6.82 ± 0.64	7.52 ± 0.19	7.03 ± 0.91	0.831
SGR	1.02 ± 0.04	0.96 ± 0.07	1.03 ± 0.02	0.98 ± 0.10	0.813
FI	9.22 ± 0.76^b^	9.52 ± 0.10^ab^	10.95 ± 0.23^a^	11.15 ± 0.42^a^	0.002
FCR	1.24 ± 0.05	1.12 ± 0.09	1.46 ± 0.02	1.66 ± 0.29	0.150
PER	2.65 ± 0.11	2.97 ± 0.24	2.25 ± 0.04	2.09 ± 0.34	0.077
K	1.65 ± 0.03	1.77 ± 0.05	1.75 ± 0.04	1.65 ± 0.05	0.074
HSI	2.23 ± 0.23	3.23 ± 1.24	2.35 ± 0.37	1.86 ± 0.28	0.122
VSI	8.02 ± 1.66	6.21 ± 0.98	7.74 ± 0.53	6.72 ± 0.52	0.103
SR	100	100	100	100	–

All values in each row with different superscripts are significantly different (*P* < 0.05).

IW: Initial weight (g); FW: Final weight (g); WG: Weight gain (g); SGR: Specific weight gain; FI: Feed intake (g); FCR: Feed conversion ratio; PER: Protein efficiency ratio; K: condition factor; HSI: Hepato-somatic index; VSI: Viscero-somatic index; SR: Survival rate (%).

### Survival rates and somatic indices

Regarding the somatic indices, the values of K, HSI, and VSI in all experimental groups were insignificantly variant amongst each other (*P* > 0.05) and no mortality was recorded in any tested group.

### Carcass biochemical analysis

Table 5 provides an illustration of the experimental fish biochemical composition. No significant differences were observed in moisture, protein, or lipid contents among groups. The fish that were fed FVW1 and FVW2 diets had numerically higher protein and lipid contents (*P* > 0.05) (14.98% and 15.00% for protein and 6.63% and 6.62% for lipid, respectively). In contrast, ash content exhibited significant variation among groups (*P* < 0.05), with the highest values observed in FVW1 (1.93%) and FVW2 (2.14%), which were significantly higher than FVW0 (1.64%) and FVW3 (1.75%).

**Table 5:** Biochemical analysis (mean ± SE) of fish Nile tilapia (*O. niloticus*) fed the experimental diets for 60 days (% wet weight).

Biochemical composition (%)	FVW0	FVW1	FVW2	FVW3	*P* value
Moisture	72.27 ± 4.30	74.31 ± 3.39	73.72 ± 0.32	74.70 ± 0.57	0.927
Protein	14.72 ± 0.84	14.98 ± 1.92	15.00 ± 0.27	14.86 ± 0.76	0.997
Lipid	5.56 ± 0.28	6.63 ± 1.00	6.62 ± 0.32	5.68 ± 0.36	0.434
Ash	1.64 ± 0.03^b^	1.93 ± 0.14^a^	2.14 ± 0.12^a^	1.75 ± 0.15^b^	0.009

All values in each row with different superscripts are significantly different (*P* < 0.05).

### Amino acids content

Table 6  summarized the amino acids profile of the experimental fish groups. The collected data illustrated an increase in most of the EAAs percent in the experimental groups relative to the control. The concentration of leucine, threonine, lysine, methionine. Arginine, and histidine percent were all significantly higher than the control (*P* < 0.05). FVW1 fish group has the highest values of leucine (6.60), threonine (4.33), lysine (7.59), arginine (6.25), valine (4.28), and histidine (2.27). In general, most EAAs followed the numerical pattern FVW1 > FVW3> FVW2 > FVW0, however, no significant differences are recorded among FVW-supplemented groups. The same trend was recorded in the NEAAs concentration as the FVW0 showed the least NEAAs concentration especially in case of aspartic acid (6.04), glycine (3.92), and proline (3.00) as their values were significantly lower compared with all other experimental groups (*P* < 0.05). Notably, FVW1 exhibited the highest numerical concentrations for several NEAAs, with aspartic acid, serine, proline, and glycine reaching statistical significance relative to the control group (*P* < 0.05).

**Table 6:** Amino acids composition (mean ± SE) of Nile tilapia (*O. niloticus*) fed the experimental diets for 60 days.

EAAs	Experimental fish groups				
	FVW0	FVW1	FVW2	FVW3	*P* value
Isoleucine	3.00 ± 0.05	3.94 ± 1.01	3.49 ± 1.32	3.86 ± 0.96	0.311
Leucine	4.65 ± 0.09^b^	6.60 ± 1.21^a^	5.88 ± 1.46^ab^	6.41 ± 1.23^a^	0.013
Threonine	2.65 ± 0.03^b^	4.33 ± 0.97^a^	3.91 ± 1.03^a^	4.17 ± 1.33^a^	0.002
Lysine	4.73 ± 0.13^b^	7.59 ± 1.13^a^	6.61 ± 1.02^a^	7.12 ± 2.01^a^	0.025
Methionine	1.38 ± 0.10^b^	2.71 ± 0.05^a^	2.42 ± 0.11^a^	2.72 ± 0.36^a^	0.017
Arginine	3.92 ± 0.08^b^	6.25 ± 1.02^a^	5.72 ± 1.00^a^	6.15 ± 1.025^a^	0.034
Valine	3.46 ± 0.012	4.28 ± 0.99	3.87 ± 0.97	4.19 ± 1.85	0.132
Phenylalanine	3.69 ± 0.09	4.28 ± 1.03	3.70 ± 0.39	3.84 ± 0.99	0.113
Histidine	1.65 ± 0.15^b^	2.27 ± 0.67^a^	2.06 ± 0.32^a^	2.24 ± 1.07^a^	0.040
Non-Essential Amino Acids (NEAAs)					
Aspartic acid	6.04 ± 2.03^b^	8.81 ± 2.46^a^	7.95 ± 2.011^a^	8.43 ± 2.55^a^	0.031
Serine	2.85 ± 1.00^b^	4.05 ± 1.03^a^	3.75 ± 0.81^ab^	3.87 ± 0.17^ab^	0.014
Glutamic acid	12.27 ± 3.02	14.62 ± 3.66	12.93 ± 3.06	13.62 ± 3.01	0.072
Glycine	3.92 ± 1.00^b^	7.92 ± 2.07^a^	7.76 ± 2.033^a^	7.68 ± 1.82^a^	0.040
Alanine	5.96 ± 1.23	6.71 ± 1.08	6.24 ± 1.085	6.59 ± 1.46	0.077
Tyrosine	2.19 ± 0.55	3.13 ± 0.97	2.76 ± 0.12	2.82 ± 0.19	0.074
Proline	3.00 ± 0.78^b^	5.06 ± 1.05^a^	5.45 ± 2.04^a^	5.12 ± 1.55^a^	0.022
Cystine	0.77 ± 0.06^b^	0.86 ± 0.04^b^	0.75 ± 0.02^b^	1.09 ± 0.08^a^	0.003
All values in each row with different superscripts are significantly different (*P* < 0.05).					

### The hematological measurements (Put Table 7 after this title)

The addition of FVW to the diets had a significant impact on the hematological parameters of Nile tilapia (Table 7). RBC counts, which measure the blood’s ability to carry oxygen, were comparatively constant across all groups, ranging from 1.41 (FVW0) to 1.56 (FVW2). Hemoglobin (Hb) levels, however, showed a more noticeable shift. In comparison to the control group (9.00) and other groups, FVW1 exhibited a significantly lower Hb (7.60). This pattern was reflected in the hematocrit (HCT) percent as it was significantly lower in FVW1 (23.63) compared to the control (28.27) and FVW2 (27.17). In comparison to the control (161.00) and FVW3 (166.00), FVW1 (179.30) and FVW2 (175.27) exhibited significantly higher MCV values. Nonetheless, no significant differences in MCH or MCHC (*P* > 0.05) were recorded. White blood cell (WBC) counts varied significantly among groups, with FVW2 showing the highest value (124.43).

Neutrophil percentages show numerical variations among groups with FVW2 and FVW3 exhibiting slightly higher values, but no statistically significant differences were observed (*P* > 0.05). Monocyte percentages varied significantly among groups, with FVW2 showing the highest value (5.67%) and FVW3 the lowest (2.67%). Eosinophil and platelet counts showed no significant differences among groups (*P* > 0.05).

**Table 7:** Complete blood count (CBC) (mean ± SE) of Nile tilapia (*O. niloticus*) fed the experimental diets for 60 days.

RBCs	FVW0	FVW1	FVW2	FVW3	*P*-Value
	1.41 ± 0.01	1.47 ± 0.08	1.56 ± 0.01	1.47 ± 0.02	0.213
Hb	9.00 ± 0.12^a^	7.60 ± 0.10^b^	8.80 ± 0.12^a^	8.50 ± 0.12^a^	0.000
HCT	28.27 ± 0.60^a^	23.63 ± 0.84^b^	27.17 ± 0.64^a^	26.30 ± 0.65^ab^	0.008
MCV	161.00 ± 0.58^b^	179.30 ± 1.65^a^	175.27 ± 0.54^a^	166.00 ± 2.52^b^	0.000
MCH	51.47 ± 0.73	55.27 ± 0.96	53.53 ± 1.79	52.27 ± 0.82	0.184
MCHC	32.70 ± 1.08	31.60 ± 1.07	31.30 ± 0.36	31.00 ± 1.00	0.427
WBCs	112.00 ± 2.65^b^	118.10 ± 2.18^ab^	124.43 ± 2.12^a^	112.67 ± 3.18^b^	0.030
Neutrophil	76.67 ± 1.45	77.17 ± 2.89	81.67 ± 1.33	81.67 ± 2.85	0.159
Lymphocytes	17.00 ± 1.53	19.33 ± 1.76	17.67 ± 1.33	15.00 ± 3.06	0.539
Monocytes	4.67 ± 0.33^a^	3.00 ± 0.33^b^	5.67 ± 0.33^a^	2.67 ± 0.33^b^	0.000
Eosinophil	1.33 ± 0.33	1.67 ± 0.33	1.67 ± 0.32	1.00±0.011	0.363
platelets	69.00 ± 4.51	83.33 ± 8.35	87.33 ± 5.84	66.33 ± 2.40	0.078

All values in each row with different superscripts are significantly different (*P* < 0.05).

RBCs: red blood cells count (10^6^/µl), Hb: hemoglobin (%), Hct: hematocrit (%); MCV: mean corpuscular volume (fL); MCH: Mean Corpuscular Hemoglobin (pg); MCHC: Mean Corpuscular hemoglobin Concentration (g/dL); WBCs: white blood cells count (10^3^/µl).

### Serum biomarkers

Dietary inclusion of FVW significantly influenced serum biochemical parameters in Nile tilapia (Table 8). FVW0 and FVW2 had moderate ALT activities (49.00 & 49.75, respectively), whereas FVW1 had the lowest (41.50) and FVW3 the highest (55.25) values. Compared to FVW0 and FVW1 (350 and 310, respectively), AST concentration was significantly higher in FVW2 and FVW3 (400.80 and 409.25, respectively). ALP activity was significantly decreased in FVW2 and FVW3 (144.50 to 141.50) relative to FVW0 and FVW1 (157.5 & 154.0). Total protein concentrations were significantly higher in all supplemented groups, ranging between 5.79 and 6.15, compared with the control group (5.07) (*P* < 0.05). Compared to the control, albumin exhibited a non-significant decrease in FVW-supplemented groups (*P* > 0.05), incontrast, these groups had significantly higher levels of globulin (4.27–4.49) relative to FVW0 (3.01) (*P* < 0.05).

**Table 8:** Serum biomarkers (mean ± SE) of Nile tilapia (*O. niloticus*) fed the experimental diets for 60 days.

ALT (U/L)	FVW0	FVW1	FVW2	FVW3	*P* value
	49.00 ± 0.91^ab^	41.50 ± 2.40^b^	49.75 ± 2.87^ab^	55.25 ± 3.75^a^	0.026
AST (U/L)	350.00 ± 5.26^b^	310.50 ± 8.41^c^	400.80 ± 12.23^a^	409.25 ± 6.97^a^	< 0.001
ALP (U/L)	157.50 ± 4.97^a^	154.00 ± 4.04^a^	144.50 ± 2.06^b^	141.50 ± 2.78^b^	0.036
Total protein (g/dL)	5.07 ± 0.08^b^	6.05 ± 0.19^a^	5.79 ± 0.10^a^	6.15 ± 0.13^a^	< 0.001
Albumin (g/dL)	2.05 ± 0.07	1.65 ± 0.18	1.53 ± 0.15	1.66 ± 0.25	0.228
Globulin (g/dL)	3.01 ± 0.13^b^	4.43 ± 0.34^a^	4.27 ± 0.08^a^	4.49 ± 0.19^a^	0.001

All values in each row with different superscripts are significantly different (*P* < 0.05).

ALT (alanine transaminase), AST (aspartate aminotransferase), and ALP (alkaline phosphatase)

### Serum digestive enzyme activities

There is a significant impact of FVW supplementation on digestive enzymes activity (Table 9). As the substitution percent increased, protease activity progressively increased until it peaked in FVW3 at 2.11. All supplemented groups showed significantly lower lipase levels (32.25–36.75) compared with the control group, which had the highest level (44.0). Amylase activity was csignificantly reduced when FVW was dietary incorporated, as FVW1–FVW3 showed 37–56% less activity than FVW0 (*P* < 0.05).

**Table 9:** Serum digestive enzymes (mean ± SE) of Nile tilapia (*O. niloticus*) fed the experimental diets for 60 days.

Proteases (U/L)	FVW0	FVW1	FVW2	FVW3	*P* value
	1.74 ± 0.04^c^	1.77 ± 0.02^c^	1.98 ± 0.02^b^	2.11 ± 0.01^a^	< 0.001
Lipase (U/L)	44.00 ± 1.08^a^	32.25 ± 2.06^b^	33.75 ± 1.11^b^	36.75 ± 2.02^b^	0.001
Amylase (U/L)	39.75 ± 1.65^a^	25.00 ± 2.48^b^	24.25 ± 1.49^b^	17.50 ± 2.75^b^	< 0.001

All values in each row with different superscripts are significantly different (*P* < 0.05).

### Intestinal histomorphology

**Fig. 1:**
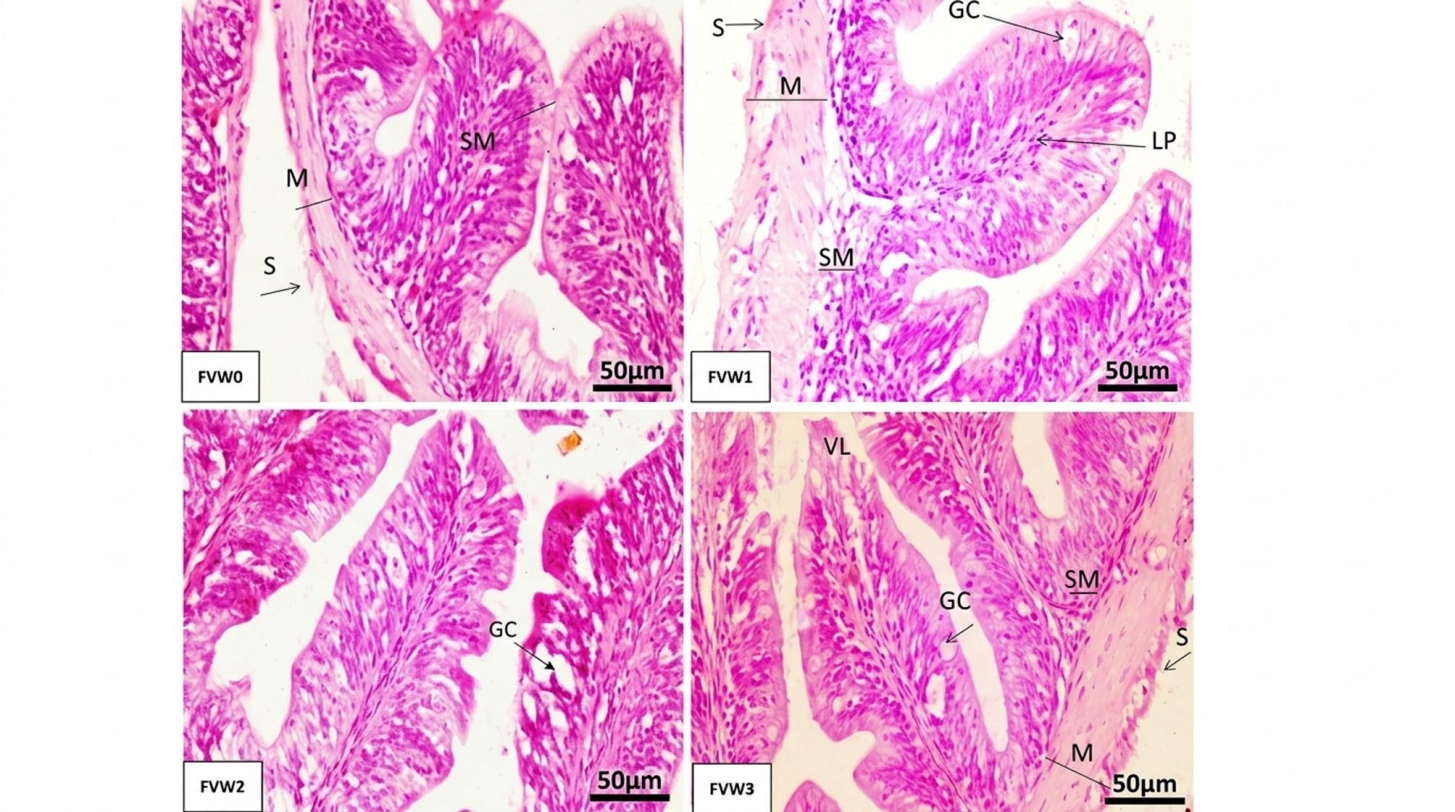
Histological structure of the distal intestine of Nile tilapia **(*Oreochromis niloticus*)** fed graded levels of fermented vegetables by- products **(FVW0-FVW3**) diets for 60 days (H&E, 40 X). All pictures showing normal intestinal architecture with intact villi, mucosa, and muscular layer. Serosa layer **(S**), muscular layer (**M)** submucosa layer (**SM**), lamina propria (**LP**) and goblet cells (**GC)**.

Histological examination of distal intestinal sections revealed normal intestinal architecture with intact mucosal layers, well-developed villi, and no histopathological lesions, indicating the safety of FVW inclusion at all tested levels (Fig. 1). The histomorphological measurements (Table 10) showed that the villi length was significantly increased in all FVW-fed groups compared to the control (125.80), with FVW2 exhibiting the maximum value (254.26), followed by FVW1 (210.76) and FVW3 (165.80) (*P* < 0.05). Villi width showed significant enhancement in FVW1 (126.53) and FVW2 (130.80) compared to control (82.84), while FVW3 showed intermediate values (109.36) (*P* < 0.05). The muscular layer thickness was significantly greater in FVW2 (17.76) compared to other groups, with FVW3 (14.35) and FVW1 (13.82) showing intermediate values, and control exhibiting the lowest thickness (11.15) (*P* < 0.05). Goblet cell count was significantly increased in FVW2 (27.00) compared to control (20.30) and FVW1 (20.60), with FVW3 showing intermediate values (22.50).

**Table 10:** Histo-morphological measurements (mean ± SE) of Nile tilapia (*O. niloticus*) fed experimental diets for 60 days.

Muscular layer (M, µm)	FVW0	FVW1	FVW2	FVW3	*P* value
	11.15 ± 9.15^c^	13.82 ± 10.95^b^	17.76 ± 2.30^a^	14.35 ± 7.55^b^	< 0.0.001
Villi Length (VL, µm)	125.80 ± 9.75^d^	210.76 ± 47.0^b^	254.26 ± 52.97^a^	165.80 ± 39.78^c^	0.000
Villi Width (VW, µm)	82.84 ± 0.36^b^	126.53 ± 6.00^a^	130.80 ± 2.00^a^	109.36 ± 1.40^ab^	0.005
Goblet Cells count (GC)	20.30 ± 1.00^b^	20.60 ± 2.50^b^	27.00 ± 2.00^a^	22.50 ± 4.50^ab^	0.049

All values in each row with different superscripts are significantly different (*P* < 0.05).

## Discussion

Solid-state fermentation (SSF) process, which turns agricultural wastes into useful bioproducts, is crucial as it enhances the nutrients bioavailability in plant-based feed stuffs through production of several microbial enzymes. In the current results, the effectiveness of solid-state fermentation in improving the nutritional value of the vegetable wastes (VW) is clear from the biochemical comparison between non fermented and fermented wastes. Crude protein content increased by about 45% after fermentation. This enhancement, along with the decrease in fiber and ash, likely played a role in the recorded physiological responses in Nile tilapia. Additionally, the experimental diets were thoughtfully designed to have the same nitrogen level and to fulfill the essential amino acid (EAA) needs of Nile tilapia. This ensures that any differences in performance could be linked to the quality and bioactivity of the fermented ingredient, not to nutrient shortages. According to Sharawy et al.^37^ and Verduzco-Oliva et al.^38^, the SSF led to a higher protein content in the fermented product. Moreover, Phinyo et al.^39^ indicated that the fermentation of soybean meal (FSBM) raised the protein content from 42% to 44% and they linked this increase to the rise in the activity of protease, cellulase, and carboxymethyl cellulase along with a high concentration of both Bacillus s*p*. and Kosakonia s*p*.

According to the current results, FVW inclusion in Nile tilapia diets may support the fish physiological status. The present results illustrated that growth performance was maintained in all FVW inclusion levels relative to the control, with numerically improved WG and SGR values observed in the FVW2 group (*P* > 0.05). The numerical growth trend and the significant enhancement in FI, in the present study, may propose a potential mitigation of ANFs as fermentation process is known to reduce them based on many previous authors^22,20^. While Phinyo et al.^39^ reported that FSBM reduced Nile tilapia growth at 30% inclusion level, the present results demonstrated that FVW could be included at the same level without negatively affecting growth performance, feed utilization, or survival rates. This discrepancy may reflect differences in the plant by-product used, as VW contain natural flavor compounds such as phenolics and organic acids that enhance feed aroma and taste^40,41^ and may enhance fish palatability, and a more diverse microbial consortium (*S. cerevisiae* & *B. subtilis*) that may more effectively degrade ANFs. These findings suggest that FVW represents a promising, cost-effective alternative to conventional fermented protein sources in Nile tilapia aquafeeds.

Although FCR and PER showed numerical variations among groups, these differences did not reach statistical significance (*P* > 0.05), suggesting that feed utilization efficiency was generally maintained across FVW inclusion levels relative to the control. Generally, growth and biometric parameters including K, HSI, and VSI showed no statistically significant differences among dietary treatments (*P* > 0.05) suggesting no evidence of major metabolic stress or organ dysfunction associated with FVW inclusion. Furthermore, 100% survival was recorded across all experimental groups, indicating that the tested diets supported fish health without adverse effects.

The carcass biochemical composition of the experimental fish showed no significant differences among groups in moisture, protein, and lipid contents (*P* > 0.05). On the other hand, the ash content significantly increased in FVW1 and FVW2 groups. The significant increment in ash content in FVW1 and FVW2 groups may reflect enhanced mineral bioavailability associated with solid-state fermentation. Desouky et al.^12^ similarly recorded no significant alterations in Nile tilapia carcass composition when fed pea peel meal at 15% and 25% inclusion levels. Furthermore, Hassan et al., (2018) observed that protein and dry matter in Nile tilapia carcass tended to decrease with increasing dietary fermented sunflower meal (SFM), while carcass lipid content showed no significant variation. Conversely, ash content increased significantly (*P* < 0.05) with increasing SFM levels in their study. According to Phinyo et al.^39^, carcass biochemical composition of Nile tilapia revealed no significant variations in protein and ash contents when fish were fed FSBM at graded inclusion levels, though a decrease in crude lipid was observed. The discrepancies between the current study and previous findings may be explained by differences in dietary protein source and processing, fermentation conditions, or experimental design.

The amino acid profile of Nile tilapia, in the current study, indicated an increase in most essential amino acids (EAAs) in fish fed FVW-supplemented diets compared with the control (FVW0). The concentrations of leucine, threonine, lysine, methionine, arginine, and histidine were significantly higher (*P* < 0.05) in FVW-fed groups, with FVW1 often exhibiting the highest numerical values. However, the response was not strictly linear across inclusion levels, suggesting that 10% FVW inclusion may represent an optimal threshold for maximizing amino acid retention in Nile tilapia, beyond which additional benefits plateau or slightly diminish. These findings highlight the effectiveness of the selected microbial consortium (*S. cerevisiae* and *B. subtilis*) in enhancing amino acid bioavailability through solid-state fermentation. The current results align with Seyoum et al.^42^, who mentioned that the EAA composition of fermented rice bran varied depending on the microbial strains used. For instance, fermentation with *Bifidobacterium longum* doubled lysine, leucine, valine, and phenylalanine content, whereas fermentation with *Limosilactobacillus fermentum*, *Lacticaseibacillus rhamnosus*, and *Lacticaseibacillus paracasei*, alone or following *Saccharomyces boulardii* reduced these EAAs. Collectively, these results underscore that the choice of microbial strains in fermentation processes is critical for determining the profile of bioactive compounds produced, which in turn may influence growth performance and immune function in the target species^43^.

Hematological parameters serve as sensitive markers for the health, nutritional condition, and detection of physiological stress in fish. The complete blood count (CBC) is an essential diagnostic tool, and evaluating blood parameters in fish has been extensively documented in aquaculture studies^44^. Additionally, changes in blood-related indicators can signify systemic disruptions in homeostasis that might impact the physiological condition of fish^45^.

In the present results, dietary incorporation of FVW induced significant alterations in several hematological parameters of Nile tilapia, suggesting adaptive responses that varied with inclusion level. White blood cell (WBC) counts varied significantly among groups, with FVW2 exhibiting the highest value, potentially indicating mild immune modulation at this inclusion level. Hemoglobin (Hb) and hematocrit (HCT) were significantly affected by diet, with FVW1 exhibiting significantly lower values relative to the control and other groups. This decrease may suggest transient alterations in oxygen-carrying capacity, potentially associated with nutrient interactions at this inclusion level. Conversely, RBC counts showed no significant variations among groups, implying that the observed changes in Hb and HCT in FVW1 were not due to reduced erythropoiesis but may reflect variations in cellular hemoglobin content. The non-linear responses observed herein where FVW1 induced pronounced changes in Hb and HCT percent while higher inclusion levels (FVW2 & FVW3) showed partial recovery align with the concept that physiological responses to dietary ingredients are not always dose-dependent. Phinyo et al.^39^ reported elevated WBC counts in Nile tilapia fed FSBM at ≥ 150 g/kg, while Desouky et al.^12^ observed no significant hematological alterations with pea peel meal inclusion in Nile tilapia diet. Discrepancies among studies may be induced by differences in the by-product composition, treatments, microbial strains, fish physiology, or experimental design. Briefly, the relationship between dietary inclusion level and physiological response is not always linear. In some cases, lower inclusion levels may induce transient adjustments without activating compensatory mechanisms, whereas higher levels may support adaptation through nutrient interactions or fermentation-derived bioactive compounds^46^.

The current study assessed the impacts of the tested diets (FVW0, FVW1, FVW2, and FVW3) on the serum biomarkers related to liver function, protein metabolism, and digestive enzymes activity. Significant variations (*p* < 0.05) were recorded for most parameters, indicating that the experimental diets induced pronounced physiological effects. AST and ALT are sensitive pointers of the hepatocyte’s integrity. ALT and AST levels were statistically the least in FVW1 group and conversely, the highest values were recorded in FVW3 group. As the fish exhibited normal growth performance, maintained high survival rates, and showed adequate levels of EAAs, the observed elevations in serum ALT and AST activities are likely an indicator to an adaptive metabolic response rather than pathological hepatocytes damage. Alkaline phosphatase (ALP) levels showed significant decline in FVW2 and FVW3 groups that may reflect alteration in metabolism or reduced hepatic ALP synthesis activity.

Total protein and globulin concentrations were significantly the lowest in FVW0 group compared to all other groups, indicating improved protein synthesis in FVW1, FVW2, and FVW3 experimental groups. Albumin concentration was insignificantly decreased across FVW-groups relative to FVW0, suggesting that the observed differences in total protein were not due to alterations in hepatic albumin production but may be due to shifts in immunoglobulin reflecting an immune-modulatory effect of the treatments. The current results are coincided with Chotolli et al.^47^ results as they tested three fruit by-products (5% of either grape, acerola, or apple meal) in Nile tilapia diets. Their results indicated that the three fruit-by products either preserved or enhanced the serum biomarkers indicating healthy fish. In summary, the observed data imply that the fermented plant ingredient (FVW) affects liver enzyme profiles reflecting modulation of liver metabolism and function due to fermentation-derived bioactive compounds.

In brief, the present findings are not inconsistent but reflect a complex, non-linear physiological adaptation to fermented feed ingredients. The apparent divergence between hematological and hepatic parameters in FVW1 needs cautious physiological interpretation. The low serum transaminases in FVW1 confirm that the observed hematological changes were not secondary to liver damage, as hepatocellular leakage would have elevated ALT and AST. Instead, the combination of low Hb and HCT with high MCV may reflect a temporary limitation in hemoglobin synthesis at the 10% inclusion level, potentially associated with altered bioavailability of iron, copper, or B-vitamins during the initial adaptation to fermented substrates.

Notably, FVW2 and FVW3 groups showed recovery in Hb and HCT to control levels, accompanied by moderate increases in ALT and AST levels (still within physiological ranges). This non-linear dose response suggests that higher FVW inclusion levels (20–30%) may provide sufficient fermentation-derived nutrients (e.g., microbial B-vitamins) with enhanced mineral bioavailability to support erythropoiesis, while the modest transaminase elevation may reflect an adaptive metabolic activity rather than a pathological symptom. Furthermore, the concurrent increase in globulin in FVW groups relative to the control with the stable albumin in all experimental groups further supports an immune-modulatory effect rather than impaired hepatic synthetic function.

Collectively, these integrated biomarker profiles indicate that FVW1 diet induced transient hematological adjustments without compromising liver integrity, whereas FVW2 and FVW3 diets supported both oxygen-carrying capacity and metabolic adaptation. This interpretation aligns with the concept that physiological responses to fermented feed ingredients are dose-dependent and may involve threshold effects for nutrient bioavailability^46^.

In the present study, digestive enzymes’ activities showed marked treatment-dependent variations. Protease activity showed a dose-dependent increase, with significant elevation observed in FVW2 and FVW3 compared to FVW0 and FVW1, that may reflect improved protein metabolism and proteolytic efficiency. Conversely, both lipase and amylase activities were significantly reduced in FVW- groups compared to FVW0 (*P* < 0.05). This decline suggests a potential redirection of metabolic resources toward protein metabolism, aligning with the observed increases in globulins and protease activity. To the best of authors’ knowledge, limited comparable data exists regarding the specific effects of fermented vegetable waste on serum digestive enzyme profiles in Nile tilapia, underscoring the novelty of the present findings. Future studies combining serum and intestinal enzyme quantification would provide a more comprehensive picture of digestive physiology in response to fermented feed.

Intestine histological examination revealed normal architecture in all FVW-supplemented groups compared with the control group. Results indicated significant improvement in intestinal histomorphology with FVW supplementation as villi length was increased significantly (*P* < 0.05) in all FVW-fed groups compared to control, with FVW2 exhibiting the maximum height, suggesting enhanced absorptive surface area. Villi width was also significantly greater in FVW1 and FVW2 groups, indicating improved structural integrity. Notably, goblet cell density showed a significant increase in FVW2 compared to control, reflecting enhanced mucosal immunity and protective mucus secretion. The muscular layer thickness was significantly elevated in FVW-fed groups, particularly FVW2, supporting improved intestinal motility and barrier function. Collectively, these histological findings demonstrate that dietary supplementation with FVW, particularly at 20% inclusion level (FVW2), significantly improves the intestinal morphological parameters, suggesting enhanced absorptive surface area, intestinal integrity, and mucosal immune function in Nile tilapia. Furthermore, these histological improvements align with the observed trends in feed utilization and growth performance, suggesting that solid-state fermentation of vegetable waste may enhance the nutrient bioavailability and intestinal wellbeing in Nile tilapia. Neves^48^ studied the effect of fermented plant-based feeds on Nile tilapia intestinal health and their results showed an increase in villi length and number of goblet cells in fish fed on fermented diet compared with the non-fermented plant-based diet, which may point to enhanced fish intestinal health and function supporting the concept that solid-state fermentation enhances intestinal health and nutrient absorption in tilapia.

Collectively, the present results support the suggestion that vegetable by-products, due to their widespread availability and nutritional value^49,50^, can be safely incorporated into Nile tilapia diets through solid-state fermentation. Furthermore, several haematological and biochemical parameters showed numerical trends without reaching statistical significance. While these observations generate hypotheses for future research, they should be interpreted cautiously within the scope of this study. Larger sample sizes or longer feeding durations may be required to detect subtle physiological effects.

## Conclusion

Fermented vegetable waste (FVW) can be safely incorporated up to 30% in Nile tilapia diets without compromising survival or growth performance. While the 10% inclusion level (FVW1) induced transient hematological adjustments, higher levels (FVW2 & FVW3) maintained oxygen-carrying capacity, enhanced immune indicators, and improved intestinal histomorphology. Serum enzyme profiles suggested a metabolic shift favoring protein utilization and the amino acids profile indicated an increase in the EAAs concentration, either significantly or not, in fish fed on FVW-supplemented diets compared with the control. Based on integrated growth, health, and histological parameters, 20–30% FVW inclusion is recommended for optimal benefits in sustainable tilapia production. Future studies are recommended to quantify exact digestibility coefficients and intestinal microbiome to further validate these findings.

## Data Availability

All data are available upon request.
